# Injury incidence in male elite youth football players is associated with preceding levels and changes in training load

**DOI:** 10.1136/bmjsem-2023-001638

**Published:** 2023-10-06

**Authors:** Tania Nilsson, Mats Börjesson, Matilda Lundblad, Andreas Ivarsson, Dan Fransson

**Affiliations:** 1 Department of Food and Nutrition and Sports Science, University of Gothenburg, Goteborg, Sweden; 2 Department of Molecular and Clinical Medicine, University of Gothenburg, Goteborg, Västra Götaland, Sweden; 3 Department of Medicine, Geriatrics, Acute Care, Sahlgrenska University Hospital, Goteborg, Sweden; 4 Department of Orthopaedics, Sahlgrenska Academy, Goteborg, Sweden; 5 School of Health and Welfare, Halmstad University, Halmstad, Sweden

**Keywords:** Adolescent, Injuries, Training, Football

## Abstract

**Objectives:**

Elite youth football players miss out on a large part of seasonal training due to injury. Limited research suggests an association between external and internal training load (TL) and injury incidence in elite youth football. This study analysed external and internal TL variables and their association with injury incidence in a group of male elite youth football players over four seasons.

**Methods:**

Measures of external and internal TL and injury incidence of 56 male elite youth football players (age 17–19 years) were collected throughout four seasons. Heart rate, session rating of perceived exertion andGlobal Positioning System (GPS) variables were analysed. Individual players’ TL during the 30 days leading up to injury was compared with 30-day injury-free control periods. Change in TL through the periods was also analysed.

**Results:**

Eighty-five injuries were included for analysis, showing that for most TL variables, the average levels were significantly lower during the period leading up to injury. Significant increases for the majority of TL variables were also found during the periods leading up to injury, while the control periods did not show any significant change.

**Conclusion:**

A lower and/or increasing average TL volume over 30 days might increase the risk of injury in male elite youth football players. Avoiding long-term drops in TL and balance increases in TL might be beneficial to reduce injury risk.

**WHAT IS ALREADY KNOWN ON THIS TOPIC**

- Injuries are common in youth football.
- The association between training load (TL) and injuries in youth football is currently not established.

**WHAT THIS STUDY ADDS**

- A low and/or increasing average TL volume during 30 days may be associated with injury risk in male elite youth football players.
- The average volume of intense accelerations over >2 m/s² during 30 days may be associated with injury in male elite youth football players rather than accelerations at lower speeds.
- Decelerations may be associated with injury incidence in male elite youth football players.
- Monitoring running at higher speeds over 24.8 km/hour might be of higher interest for youth football teams compared with running at lower speeds

**HOW THIS STUDY MIGHT AFFECT RESEARCH, PRACTICE OR POLICY**

- The results of this study indicate that periods with low and/or increasing average TL volume can be periods when players have an increased risk of injury. A well-developed training exposure and a balanced progression of TL could potentially reduce the injury risk.

## Introduction

Injuries affect the youth football player’s development, health and possibly burden club/parental finances and healthcare systems.^1^ One potential modifiable risk factor for injuries in senior football is rapid increases in external training load (TL) (ie, the physical work to which the athlete is exposed^2^ such as distance performed at different speeds),^3^ and internal TL (ie, the physiological and psychological response to external^2^ TL such as session rate of perceived exertion (S-RPE)).^4^ Worth noting is that the majority of the studies conducted on TL and injuries have shown methodological problems, and no study has yet been able to prove a causal relationship between TL and injuries.^5 6^

The association between external TL and injury risk in elite youth football has been studied by Bacon *et al*^7^ who found that weekly averages of total distance (TD) but not high-speed running (HSR) were associated with higher overuse injury risk. However, the study did not clarify which speed zone HSR represented. Only 16 injuries were included, and they used a small group of participants (n=41).^7^ The association between internal TL and injury risk in elite youth football has been studied by Brink *et al*^8^ who found that S-RPE was associated with injuries. Still, other studies found no association.^5 9 10^ Heart rate (HR) during exercise and its association with injury in youth football players is, to our knowledge, lacking in the scientific literature.

The acute:chronic workload ratio (ACWR) has been used in previous studies to calculate correlations between TL and injuries,^11 12^ but this research has been questioned due to methodological errors such as the risk of type I errors.^6^ The available evidence exploring TL and its association with injury risk is unreliable for providing practical recommendations on using TL to reduce injury risk in youth football players.^6^ Therefore, this study aimed to enhance the understanding and contribute to the research field by focusing on TL and its potential association with injury risk. The study aimed to achieve this by examining the absolute values and capturing changes in external and internal TL variables over a 30-day period and their potential association with injury incidence, in a group of Swedish male elite youth football players.

## Methods

### Participants

The study was designed as an observational cohort study. Data were collected from 56 male elite youth football players aged 17–19 years from one football club in Sweden’s first division for this age group (stature: 181.0±7.8 cm, body mass: 71.2±7.2 kg) during the seasons 2018–2021. Baseline data were collected at player inclusion every season. All playing positions (23 defenders, 29 midfielders, 4 attackers) were represented except goalkeepers due to their different nature of activity. Two players (4 %), 17 players (30 %) and 37 players (66 %) participated in three, two and one seasons, respectively. For a power calculation, we decided that an effect of 0.2 for the relationship between the type of sequence and the level of the outcome variable would be of practical interest.^13^ The power calculation, using the simr package in r (V.4.3.1), showed that a minimum of 40 participants was needed to achieve sufficient power (0.80) for the parameter of interest.

### Training load quantification

The following variables were collected and analysed: average HR and S-RPE, total distance (TD: >0 km/hour), high-intensity running distance (HIRd: 15–19.79 km/hour), high-speed running distance (HSRd: 19.8–24.79 km/hour), sprint distance (Sd: 24.8–29.79 km/hour), maximal sprint distance (Msd: >29.8 km/hour), accelerations (Acc: >0.50 m/s²), intense accelerations (IA: >2.00 m/s²), very intense accelerations (VIA: >3.00 m/s²), decelerations (Dec: <−0.50 m/s²), intense decelerations (ID: <−2.00 m/s²) and very intense decelerations (<−3.00 m/s²). The variables defined above were selected due to their relevance to football TL and potential injury.^3 14^

GPS-derived TL and average HR were quantified during all outfield training sessions and games using 10 Hz GPS and heart rate units (Polar Team Pro; Polar Electro, Kempele, Finland). This specific unit has a high to extremely high accuracy (ICC=0.63–0.99) in different speed parameters and an interunit reliability of 0.62–0.99 in ICC values.^15^ The heart rate measure has been validated and used in previous research.^16 17^ The unit was placed on a chest strap, and the same player wore the same unit at all sessions and games. The time for minimum effort duration was set to 1 s, and data sets were verified for the number of satellites connected (mean >11) and horizontal dilution of precision (mean <1.2) before being included in the analysis.^18^ Rating of perceived exertion (RPE) was collected after every training session (including gym sessions) and games by asking the players: ‘How was your workout?’. The players answered on a modified category scale from 0 min to 10 30 min after the session. S-RPE was calculated by multiplying the RPE value by the duration of each training session or game.^19^

All TL variables were analysed by dividing individual players’ data into a 30 days leading up to injury, including the day of injury, and comparing this to a 30-day control period. This model was chosen to compare the individual players with themselves, enabling an individual aspect through the statistical analysis. The control period was 30 days without any absence from training due to injury, illness or vacation. The control periods were spread throughout the year, including preseasons and competitive seasons. In cases of missing data (ie, the player forgot the GPS, GPS was out of function, or S-RPE was not collected), an average based on the individual player’s game or training values throughout the season was used. This approach has been used in previous research.^4 20^

### Injury definition

The team’s medical staff recorded player exposure and time-loss injuries (ie, when a player was absent from future football participation)^21^ using a standard exposure and injury form. Players with an existing injury at the start of the study were included in the study, but their particular injury and the exposure details were not included in the injury statistics until the player could take part in full training and game.^21^

### Statistical analysis

Results are presented in figures and tables. Descriptive statistics are presented as mean and SD. Injury incidence was calculated as the number of injuries per 1000 hours of exposure in training and/or games, as is standard.^21^

Due to the nested data, we applied a three-level model specification (eg, see Mplus syntax in online supplemental file 1). Within all analyses, days (level 1) were nested in 30-day periods (level 2). The periods were, in turn, nested in individuals (level 3). To analyse if there was any association between the type of periods (ie, the period leading up to an injury with a control period) and the 30-day average in the different TL variables, we included a dichotomous covariate on level 2 for all the analyses. To analyse if there were any changes in the internal and external TL variables during the periods, the variable days (ie, ranging from 30 to 0) were regressed on the TL variables on level 1.

10.1136/bmjsem-2023-001638.supp1 Supplementary data

The When-to-Worry-and-How-to-Avoid-the-Misuse-of-Bayesian-Statistics (WAMBS) checklist was used to guide the report of the analytical strategy and the results. All statistical analyses were estimated using Bayesian estimation in Mplus V.8.0. One of the advantages of the Bayesian estimator is, in comparison to the frequentist estimator, the increased likelihood of producing reliable estimates with small sample sizes.^22^ For the analyses, we performed Markov Chain Monte Carlo simulation procedures with a Gibbs sampler with 100 000 iterations. Four Markov chains were implemented for each of the specified parameters. We assessed chain convergence by applying the Gelman and Rubin convergence diagnostic (this process is described in the Mplus manual). We, however, applied a stricter convergence criterion than the default setting (0.01 instead of 0.05). For all analyses, the Gelman and Rubin diagnostic indicated adequate convergence for each of the four chains. In the next step, we visually inspected the trace plots for each parameter. In this inspection, all four chains showed adequate convergence for each parameter. To validate that convergence was obtained and ruled out potential errors related to local convergence, we ran the analyses again using 200 000 interactions. The Gelman and Rubin convergence diagnosis indicated convergence for all parameters in the analyses. Because of the novel design, we decided to rely on default prior settings (N(0, 10^10^)) of the software for the variance on all parameters (ie, intercepts, factor loadings and regression).

Model fit was, for all models, assessed using the posterior predictive *p* (PP*p*) value and it is accompanying 95% CI.^23^ We estimated credibility intervals (CI) for all variables within the models. We rejected the null hypothesis if the 95% CI did not include zero,^24^ and then considered the results as statistically significant.

### Patient and public involvement statement

Players were involved from the start of the measures. The players participated in their own team’s normal training activities each week. The participating clubs will receive a written report on the study results.

## Results

### Exposure and incidence of injury

The players completed, on average, 130 individual football sessions and 31 games per year and performed one strength session per week. Missing data represented 3% of the GPS variables, 3% of the HR variable and 2% of the S-RPE variable. Given the small amount of missing data, the impact was considered negligible. Ninety-five injuries were recorded during the four seasons. The incidence of injury was estimated to be 4.2 injuries per 1000 exposure hours and 12.2 and 3.0 injuries per 1000 exposure hours for games and training, respectively. Traumatic injuries (injury with sudden onset and known cause^25^ accounted for 71% (n=60) and overuse injuries (injury with insidious onset and no known trauma)^25^ represented 29% (n=25) of all injuries. Ten injuries were excluded from further analysis due to a major lack of TL data. In summary, 85 injuries were used for further analysis.

### TL and injury

All models specified to analyse the association between the periods, leading up to injury and the control periods and average level on the TL variables, showed a good fit to the data. The three-level analyses showed significant negative associations between the periods leading up to injury and all TL variables except ID, HIRd and HSRd. More specifically, the average level on all variables was consistently lower during the periods, leading up to injury compared with the control periods (table 1). For all parameter estimates, see online supplemental file 2.

10.1136/bmjsem-2023-001638.supp2 Supplementary data

**Table 1:** Average levels for TL variables during the control periods compared with the periods leading up to injury for 56 male elite youth football players aged 17–19

Variable	B (95% CI)	Control period M (SD)	The period leading up to the injury M (SD)
S-RPE (au)	0.26 (−0.49 to 02)	351.04 (12.33)	326.19 (9.76)
Average HR (bpm)	0.28 (−0.49 to −0.05)	71.73 (3.11)	64.38 (2.25)
TD (m)	0.24 (−0.44 to −0.03)	2851.3 (134.7)	2523.3 (107.3)
ACC (ne)	0.23 (−0.43 to −0.02)	312.45 (15.22)	278.77 (11.79)
IA (ne)	0.28 (−0.51 to −0.04)	34.42 (1.81)	30.00 (1.54)
VIA (ne)	0.34 (−0.69 to −0.02)	6.35 (0.54)	5.40 (0.36)
DEC (ne)	0.24 (−0.44 to −0.04)	350.33 (16.80)	309.82 (12.99)
ID (ne)	0.22 (−0.56 to .04)	32.84 (2.21)	28.73 (1.70)
VID (ne)	0.32 (−0.54 to −0.08)	7.08 (0.49)	5.96 (0.39)
HIRd (m)	0.21 (−0.44 to .03)	237.72 (12.38)	212.53 (12.19)
HSRd (m)	0.21 (−0.44 to .03)	105.57 (6.81)	93.13 (6.04)
Sd (m)	0.28 (−0.52 to −0.02)	35.85 (3.12)	29.46 (2.78)
Msd (m)	0.31 (−0.55, −0.07)	4.20 (0.75)	3.06 (0.42)

Acc, accelerations (>0,5 m/s2); au, arbitrary units; B, unstandardised beta coefficient; bpm, beats per minute; CI, credibility interval; Dec, decelerations (< −0); HIRd, high-intensity running distance (15−19.79 km/hour); HSRd, high-speed running distance (19.8−24.79 km/h); IA, Intense accelerations (>2 m/s²); ID, intense decelerations (<−2 m/s²); m, metres; Msd, maximal sprint distance (>29.8 km/h); ne, number of efforts; PPp, posterior predictive p-value; Sd, Sprint distance (24.8–29.9 km/h); S-RPE, session rate of perceived exertion; TD, total distance (>0 km/h); VIA, very intense accelerations (>3 m/s²); VID, very intense decelerations (<−3 m/s²).

All models specified to analyse if a change in the TL variables during the 30-day periods fits the data well. An average of 20.6 and 19.3 training sessions was conducted during the control periods and periods leading up to injury, respectively. Significant positive changes on all TL variables, except Acc, indicated an increase in TL during the periods, leading up to injury (table 2). There was no significant change in any TL variable during the control periods. For all parameter estimates, see online supplemental file 2.

**Table 2:** Average change estimates (with 95% CI) for the variables in the control periods and the periods leading up to injury for 56 male elite youth football players aged 17–19 years old

Variable	Change over the 30 days leading up to injury (B, 95% CI)	Changes over the 30 days control period (B, 95% CI)
S-RPE (au)	2.30 (0.97 to 3.62)	0.90 (2.66 to 0.80)
Average HR (bpm)	0.81 (0.52 to 1.09)	0.22 (0.60 to 0.15)
TD (m)	0.38 (0.24 to 0.51)	0.19 (0.16 to 0.20)
ACC (ne)	3.67 (2.28 to 5.03)	0.42 (2.25 to 1.35)
IA (ne)	0.31 (0.13 to 0.51)	0.03 (0.28 to 0.21)
VIA (ne)	0.04 (0.01 to 0.08)	0.01 (0.09 to 0.06)
DEC (ne)	4.03 (2.52 to 5.52)	0.64 (2.65 to 1.31)
ID (ne)	0.26 (0.02 to 0.50)	0.01 (0.35 to 0.36)
VID (ne)	0.09 (0.05 to 0.13)	0.02 (0.04 to 0.08)
HIRd (m)	3.21 (1.74 to 4.67)	0.61 (1.38 to 2.53)
HSRd (m)	1.26 (0.58 to 1.95)	0.32 (0.57 to 1.19)
Sd (m)	0.61 (0.32 to 0.90)	0.02 (0.41 to 0.36)
Msd (m)	0.09 (0.04 to 0.14)	0.02 (0.10 to 0.05)

Acc, accelerations (>0); au, arbitrary units; B, unstandardised beta coefficient; bpm, beats per minute; CI, credibility interval; Dec, decelerations (<−0); HIRd, high-intensity running distance (15−19.79 km/hour); HSRd, high-speed running distance (19.8−24.79 km/h); IA, intense accelerations (>2 m/s²); ID, intense decelerations (<−2 m/s²); m, metres; Msd, maximal sprint distance (>29.8 km/h); ne, number of efforts; Sd, Sprint distance (24.8–29.9 km/h); S-RPE, session rate of perceived exertion; TD, total distance (>0 km/h); VIA, very intense accelerations (>3 m/s²); VID, very intense decelerations (<−3 m/s²).

## Discussion

The main findings of this study were that external and internal TL is associated with injury incidence in male elite youth football. The average level of all variables, except intense ID, HIRd and HSRd, were significantly lower during the periods leading up to injury in comparison to the control periods. In addition, significant increases in all TL variables except accelerations (Acc) were found during the periods leading up to injury, while no significant change in any TL variable was found during the control periods. In summary, the results of the present study indicate that periods with low and/or increasing average TL volume might be periods where players are at a higher risk of sustaining an injury.

### Injury incidence

Injury incidence was estimated to be 4.2 injuries per 1000 hour of exposure, considerably lower compared with previous findings on elite football players of the same age.^9 10^ Injury incidence can be affected by factors such as methodological differences in injury definition or reporting systems,^26^ levels of play, player age and previous injury.^3 27^

### TL and injury

**Figure 1:**
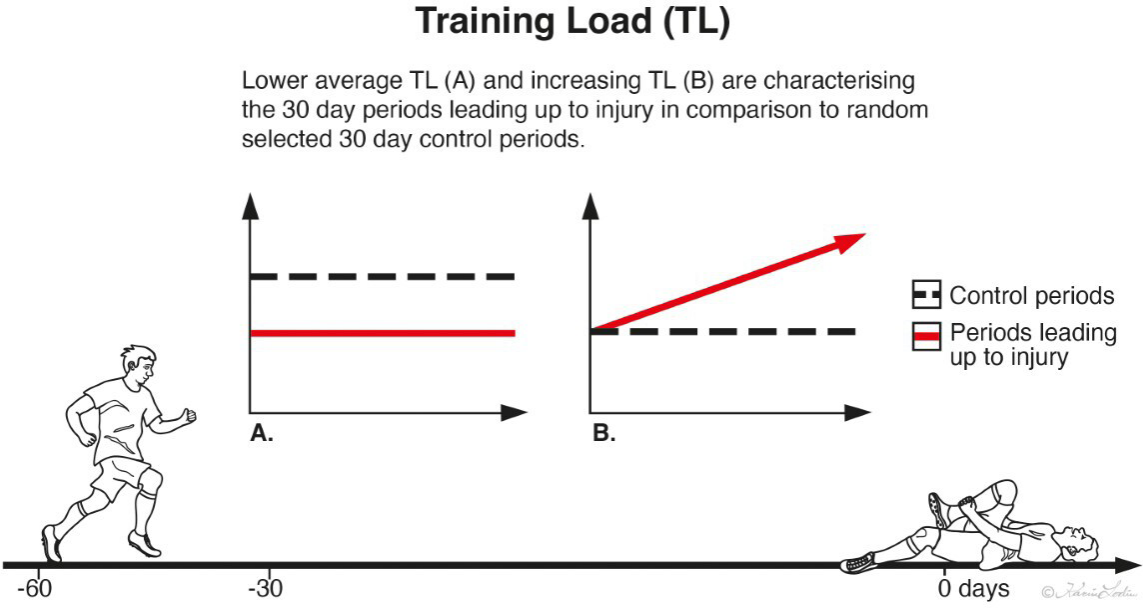
Association between training load and subsequent injury.

Most TL variables had a lower average volume during the periods leading up to injury, while the control periods had a higher average volume (figure 1). The number of training sessions was relatively equal between the compared periods. Potentially, a high average TL volume over time (as during the control periods) in combination with adequate recovery might increase the strength of the tissues, possibly leading to a better tolerance against increasing TL and subsequent injury.^9 28^ An excessive overload or a rapid progression in TL can on the other hand lead to suboptimal adaptations where the strength of the tissues involved might be exceeded, potentially increasing the risk of injury or causing tissue damage.^29 30^ Interestingly, the majority of TL variables in this study showed an increase in average volume during the periods leading up to injury (figure 1).

Running at higher speeds puts higher physiological stress on the body than running at lower speeds.^28^ In this study, the average running volume at lower speeds under 24.79 km/hour (HIRd and HSRd) was equal between control periods and periods leading up to injury. However, for running speeds over 24.8 km/hour (Sd and Msd), the average volume was lower during the periods leading up to injury. In a recent paper, Edouard *et al*^31^ discussed that optimal exposure to maximal or near-maximal running velocity might be a protective factor for hamstring injuries and that a lack of regular preparatory sprint training might lead to a higher risk of sprint-related injuries. The authors suggested that athletes should be exposed to sprinting regularly to avoid a long-term drop in sprinting volume and potential injury.^31^ This approach can be adapted to the present study results as well. Monitoring running at higher speeds might, therefore, interest youth football teams.

The results from the present study also indicate that changes in volume in intense accelerations (IA and VIA) over >2 m/s² should be of particular interest to monitor rather than accelerations at lower speeds, since the variable Acc that includes accelerations in lower speeds was the only variable not showing an association between change in volume during 30 days and injury incidence. Accelerating at higher speeds puts higher physiological demands on the body than accelerating at lower speeds,^32^ which may be why increasing volumes of accelerations at higher speeds is associated with injury in this study.

Dec have been associated with injury risk in senior football,^14^ and future research has been requested to evaluate its association with injury in youth football.^33^ Our results show an increasing average volume in Dec during the periods leading up to injury compared with the control periods, which indicates that Dec is associated with injury risk in elite youth football players.

Previous literature investigating the association between S-RPE and risk of injury for youth football players shows low support when using the acute-chronic workload ratio.^5 9 10^ HR has previously been associated with injuries in elite senior football^34^ but not in elite youth football. In the present study, S-RPE and average HR were associated with injury risk since both variables had a lower and increasing average volume during the periods leading up to injury compared with the control periods.

Future multifactorial research of high quality is required to further assess the relationships between measures of TL and injury risk for football players. Researchers should also consider examining injuries and changes over time in TL for shorter periods than 30 days.

### Clinical implications

Evaluating TL from an individual perspective can identify periods with a low and/or increasing average TL volume where players might have an increased risk of injury. A well-developed training exposure and a balanced progression of TL can be beneficial to reduce injury risk. To deal with this, every athlete should be evaluated individually and monitored regularly, and the data should be compared longitudinally.

### Limitations

We acknowledge that there are major methodological challenges in conducting this type of research.^6 35^ Several previous studies have used the ACWR to calculate correlations between TL and injuries and the possibilities of predicting injuries.^11 12^ These studies have been questioned due to methodological errors,^5 15^ such as the risk of type I errors due to calculation associations by using multiple combinations.^6^ Other methodological errors considered are that the ACWR is used to control for chronic workload (the denominator variable), which is assumed to influence the acute workload (the numerator). Still, researchers have shown that the ACWR fails to normalise the numerator for the denominator.^36^ Impellizzeri and colleagues describe this problem well in the study Training load and Its Role in Injury Prevention, Part 2: Conceptual and Methodologic Pitfalls^6^: *This failure adds unnecessary ‘‘noise,’’ increases the risk of artifact and makes the results difficult to interpret, as the practitioner cannot determine whether the acute or chronic TL is driving the ACWR*.^6^ In the present study, we wanted to investigate TL’s association with injuries by not using a ratio but instead using a multilevel model recommended by Windt *et al*.^37^ We investigated absolute values and captured changes in TL over 30 days. Still, we did not look at cumulative values or day-to-day fluctuation in TL, which can be considered a limitation of the study. Recent studies have also suggested that non-linear models might be beneficial in discovering relationships between TL and injury.^35^ A more consistent methodology for TL and injury risk studies can enable better comparability and reproducibility within this research area in the future.

Confounding factors can affect whether a player gets injured, such as the possibility and quality of recovery between training sessions,^29^ the implementation of injury prevention programmes^6^ or previous injury.^27^ This information was unavailable and, therefore, not included in the analysis in this study. Also, we did not have enough data to perform separate analyses for traumatic and overuse injuries. This is a limitation given the potential risk factors for these two types of injuries.

Sample size and data from only one club can be a study limitation. Another limitation is that the club recorded the data, and it is unknown whether the practitioners adapted TL to protect players. We acknowledge that the diversity was limited to this population, excluding male youth football players at grassroots levels and those from other geographical regions in Sweden.

## Conclusion

Injury is a complex and dynamic outcome affected by many different risk factors, often with no predictable pattern,^38^ which makes the goal of decreasing injury risk hard to reach. We cannot prove a causal relationship between TL and injury. Still, this study suggests that periods with low and/or increasing average TL volume might be periods when players have an increased risk of injury. Therefore, evaluating TL from an individual perspective can identify periods when players potentially have an increased risk of injury, representing one piece of the injury prevention puzzle.^39^ Practitioners may use these indicators in TL combined with their clinical experience and knowledge of physiology and basic training principles to decrease injury risk.

## Data Availability

Data are available upon reasonable request. Except for the data presented in the study, more information about injury descriptions or raw data are the property of the club and therefore only available to the researchers, the players and the club only.
