# Supplementary material for: Injury incidence in male elite youth football players is associated with preceding levels and changes in training load
Source: BMJ Open Sport Exerc Med. 2023 Oct 6;9(4):e001638. doi: 10.1136/bmjsem-2023-001638 (PMC10565147; doi:10.1136/bmjsem-2023-001638)
Supplement: Supplementary data [file bmjsem-2023-001638supp001.pdf]

```
TITLE: Syntax for the three-level analysis with Sequence Type as co-variate on level 2.
DATA:
  FILE IS "Study.dat";
VARIABLE:
NAMES ARE ID Clus Type N_Time !ID = Level 3 Clus = Level 2 Type = Sequence Type
S_RPE HR TD ACC IA VIA DEC ID VID HIRd
HSRd Sd Msd;
 USEVARIABLES ARE
  Type
  S_RPE
  ;
MISSING ARE ALL (-111);
CLUSTER = ID Clus;
BETWEEN (Clus) = Type;
ANALYSIS:
TYPE IS THREELEVEL RANDOM;
ESTIMATOR = BAYES;
Bconvergence = .01;
Biterations = 40000 (10000);
Processor = 4;
Chains = 4;
thin = 10;
BSEED = 1234;

MODEL:
%BETWEEN ID%
%BETWEEN Clus%
S_RPE ON Type;
%WITHIN%

OUTPUT: STDYX
```

```
CINTERVAL TECH1 TECH4;

PLOT:

TYPE IS PLOT2;
```

```
TITLE: Syntax for the three-level analysis with time as a co-variate on level 1.
DATA:
  FILE IS "Study.dat";
VARIABLE:
NAMES ARE ID Clus Type N_Time !ID = Level 3 Clus = Level 2 Type = Sequence Type
S_RPE HR TD ACC IA VIA DEC ID VID HIRd
HSRd Sd Msd;
 USEVARIABLES ARE
  N_Time
  S_RPE
  ;
MISSING ARE ALL (-111);
CLUSTER = ID Clus;
Within  = N_Time;
ANALYSIS:
TYPE IS THREELEVEL RANDOM;
ESTIMATOR = BAYES;
Bconvergence = .01;
Biterations = 40000 (10000);
Processor = 4;
Chains = 4;
thin = 10;
BSEED = 1234;

MODEL:
%BETWEEN ID%
%BETWEEN Clus%
%WITHIN%
S_RPE ON N_Time;
OUTPUT: STDYX
CINTERVAL TECH1 TECH4;
```

PLOT:

TYPE IS PLOT2;
