# Supplementary material for: Injury incidence in male elite youth football players is associated with preceding levels and changes in training load
Source: BMJ Open Sport Exerc Med. 2023 Oct 6;9(4):e001638. doi: 10.1136/bmjsem-2023-001638 (PMC10565147; doi:10.1136/bmjsem-2023-001638)
Supplement: Supplementary data [file bmjsem-2023-001638supp002.pdf]

Table 3 Supplementary information

	**S-RPE (au)**	**Average HR (bpm)**	**TD (m)**	**ACC (ne)**	**IA (ne)**	**VIA (ne)**	**DEC (ne)**	**ID (ne)**	**VID (ne)**	**HIRd (m)**	**HSRd (m)**	**Sd (m)**	**Msd (m)**
**Fixed effects level 2**													
sequence type	-0.26 [-0.49, -0.02]	-.0.28 [-0.49, -0.05]	-0.24 [-0.44, -0.03]	-0.23 [-0.43, -0.02]	-0.28 [-0.51, -0.04]	-0.34 [-0.69, -0.02]	-0.24 [-0.44, -0.04]	-0.25 [-0.56, 0.04]	-0.32 [-0.54, - 0.08]	-0.21 [-0.44, 0.03]	-0.21 [-0.44, 0.03]	-0.28 [-0.52, - 0.02]	-0.31 [-0.55, -0.07]
**Ramdom variances level 1**													
Variance	96728.70 [92657.60, 100997.02]	4502.79 [4212.67, 4702.00]	100617.70 [96424.80, 105094.70]	NA [99728.31, 108714.92]	1885.06 [1805.77, 1968.58]	137.63 [131.82, 143.72]	NA [119593.43, 130375.34]	3081.62 [2951.53, 3218.79]	96.72 [92.67, 101.01]	NA [113822.89, 124086.77]	25377.09 [24304.97, 26497.56]	4522.20 [4331.22, 4722.48]	147.45 [141.23, 154.04]
**Random variances level 2**													
Residual variance	2454.70 [1166.66, 4238.50]	146.39 [75.76, 247.03]	4080.00 [2383.40, 6376.60]	4729.83 [2794.96, 7427.22]	50.80 [24.03, 89.65]	1.68 [0.29, 3.79]	5595.11 [3310.91, 8783.95]	47.74 [12.77, 98.43]	2.82 [1.38, 4.90]	3424.08 [1634.83, 5928.64]	746.48 [364.58, 1308.14]	106.18 [44.62, 197.71]	3.70 [1.62, 6.68]
**Random variances level 3**													
Variance	897.34 [123.05, 2469.92]	76.74 [15.51, 189.47]	771.30 [49.30, 2703.60]	1570.93 [206.34, 4336.35]	39.85 [11.25, 90.49]	3.45 [1.56, 6.82]	1924.17 [278.44, 5213.49]	50.27 [15.32, 112.55]	3.61 [1.53, 7.26]	1616.93 [196.46, 4524.18]	738.12 [236.47, 1615.29]	163.89 [69.01, 332.75]	6.76 [3.23, 13.06]
**Model fit indices**													
PPp	0.49	0.48	0.49	0.49	0.49	0.48	0.49	0.48	0.49	0.49	0.48	0.47	0.48
95 % Confidence Inverval	[-10.91, 11.99]	[-11.11, 11.84]	[-11.01, 12.17]	[-10.64, 11.72]	[-11.12, 12.03]	[-10.55, 12.07]	[-10.67, 11.73]	[-11.05, 11.64]	[-10.87, 12.00]	[-10.89, 11.69]	[-10.90, 11.16]	[-9.79, 11.56]	[-10.67, 11.94]

PPp: Posterior Predictive p-value, B: Unstandardized Beta Coefficient, CI: Credibility interval, SD: standard deviation, au: arbitrary units, bpm: beats per minute, m: meters, ne: number of efforts, S-RPE: Session rate of perceived exertion, TD: Total distance (>0 km/h), Acc: Accelerations (>0,5 m/s2), IA: Intense accelerations (>2 m/s^2), VIA: Very intense accelerations (>3 m/s^2), Dec: Decelerations (< -0,5 m/s2), ID: Intense decelerations (< -2 m/s^2), VID: Very intense decelerations (< -3 m/s^2), HIRd: High-intensity running distance (15 km/h - 19,79 km/h), HSRd: High-speed running distance (19,-24,79 km/h), Sd: Sprint distance (24,8 – 29,9 km/h), Msd: Maximal sprint distance (> 29,8 km/h).

Table 4 Supplementary information

	S-RPE (au)	Average HR (bpm)	TD (m)	ACC (ne)	IA (ne)	VIA (ne)	DEC (ne)	ID (ne)	VID (ne)	HIRd (m)	HSRd (m)	Sd (m)	Msd (m)
**Fixed effects level 1**													
time	2.30 [0.97, 3.62]	0.81 [0.52, 1.09]	0.38 [0.24, 0.51]	3.67 [2.28, 5.03]	0.31 [0.13, 0.51]	0.04 [-0.01, 0.08]	4.03 [2.52, 5.52]	0.26 [0.02, 0.50]	0.09 [0.05, 0.13]	3.21 [1.74, 4.67]	1.26 [0.58, 1.95]	0.61 [0.32, 0.90]	0.09 [0.04, 0.14]
**Ramdom variances level 1**													
Residual variance	97971.35 [92881.33, 103605.96]	4493.19 [4258.53, 4751.90]	99636.30 [94430.20, 105339.90]	NA [98952.13, 110412.74]	1971.01 [1867.98, 2083.74]	124.62 [118.10, 131.77]	NA [117503.89, 131089.09]	3158.97 [2992.68, 3339.63]	90.01 [85.33, 95.13]	NA [111492.28, 124365.02]	25908.32 [24555.48, 27381.14]	4553.49 [4314.40, 4812.91]	120.68 [114.37, 127.56]
**Random variances level 2**													
Variance	2670.24 [1006.66, 5183.00]	169.06 [77.23, 307.03]	4830.00 [2630.40, 8191.50]	5539.88 [2972.54, 9407.94]	77.88 [35.71, 139.17]	2.82 [0.73, 5.88]	6352.78 [3341.26, 10861.82]	88.76 [35.30, 166.44]	3.92 [1.68, 7.37]	5269.93 [2513.01, 9228.07]	1170.67 [526.95, 2096.72]	121.28 [26.94, 278.93]	2.19 [0.31, 5.44]
**Random variances level 3**													
Variance	615.40 [27.68, 2533.13]	39.41 [1.61, 154.24]	564.70 [20.40, 2855.50]	990.93 [41.02, 4190.43]	18.32 [0.74, 72.36]	1.32 [0.08, 4.07]	1291.14 [57.11, 5140.47]	16.31 [0.61, 71.43]	2.30 [0.17, 6.55]	1125.49 [39.74, 5182.58]	381.13 [16.18, 1411.12]	151.72 [16.54, 383.91]	3.30 [0.73, 7.63]
**Model fit indices**													
PPp	0.47	0.48	0.47	0.47	0.48	0.47	0.46	0.47	0.46	0.47	0.48	0.47	0.46
95 % Confidence Inverval	[-10.36, 11.39]	[-10.46, 11.80]	[-10.29, 11.69]	[-10.40, 11.19]	[-10.82, 10.98]	[-10.42, 11.34]	[-10.45, 11.78]	[-10.31, 11.73]	[-10.18, 11.33]	[-10.29, 11.80]	[-10.05, 11.10]	[-10.44, 11.75]	[-10.19, 11.58]

PPp: Posterior Predictive p-value, B: Unstandardized Beta Coefficient, CI: Credibility interval, SD: standard deviation, au: arbitrary units, bpm: beats per minute, m: meters, ne: number of efforts, S-RPE: Session rate of perceived exertion, TD: Total distance (>0 km/h), Acc: Accelerations (>0,5 m/s2), IA: Intense accelerations (>2 m/s^2), VIA: Very intense accelerations (>3 m/s^2), Dec: Decelerations (< -0,5 m/s2), ID: Intense decelerations (< -2 m/s^2), VID: Very intense decelerations (< -3 m/s^2), HIRd: High-intensity running distance (15 km/h - 19,79 km/h), HSRd: High-speed running distance (19,-24,79 km/h), Sd: Sprint distance (24,8 – 29,9 km/h), Msd: Maximal sprint distance (> 29,8 km/h).
